# Evidence of Synaptic and Neurochemical Remodeling in the Retina of Aging Degus

**DOI:** 10.3389/fnins.2020.00161

**Published:** 2020-03-18

**Authors:** Lily Y.-L. Chang, Alvaro O. Ardiles, Cheril Tapia-Rojas, Joaquin Araya, Nibaldo C. Inestrosa, Adrian G. Palacios, Monica L. Acosta

**Affiliations:** ^1^School of Optometry and Vision Science, The University of Auckland, Auckland, New Zealand; ^2^Centro Interdisciplinario de Neurociencia de Valparaíso, Universidad de Valparaíso, Valparaíso, Chile; ^3^Department of Cell and Molecular Biology, Faculty of Biological Sciences, Center for Aging and Regeneration, Pontificia Universidad Católica de Chile, Santiago, Chile; ^4^New Zealand National Eye Centre, The University of Auckland, Auckland, New Zealand; ^5^Faculty of Medical and Health Sciences, Centre for Brain Research, The University of Auckland, Auckland, New Zealand; ^6^Brain Research New Zealand – Rangahau Roro Aotearoa, Auckland, New Zealand

**Keywords:** retina remodeling, amyloid protein, aging, Alzheimer’s disease, *Octodon degus*

## Abstract

Accumulation of amyloid-beta (Aβ) peptides is regarded as the hallmark of neurodegenerative alterations in the brain of Alzheimer’s disease (AD) patients. In the eye, accumulation of Aβ peptides has also been suggested to be a trigger of retinal neurodegenerative mechanisms. Some pathological aspects associated with Aβ levels in the brain are synaptic dysfunction, neurochemical remodeling and glial activation, but these changes have not been established in the retina of animals with Aβ accumulation. We have employed the *Octodon degus* in which Aβ peptides accumulated in the brain and retina as a function of age. This current study investigated microglial morphology, expression of PSD95, synaptophysin, Iba-1 and choline acetyltransferase (ChAT) in the retina of juvenile, young and adult degus using immunolabeling methods. Neurotransmitters glutamate and gamma-aminobutyric acid (GABA) were detected using immunogold labeling and glutamate receptor subunits were quantified using Western blotting. There was an age-related increase in presynaptic and a decrease in post-synaptic retinal proteins in the retinal plexiform layers. Immunolabeling showed changes in microglial morphology characteristic of intermediate stages of activation around the optic nerve head (ONH) and decreasing activation toward the peripheral retina. Neurotransmitter expression pattern changed at juvenile ages but was similar in adults. Collectively, the results suggest that microglial activation, synaptic remodeling and neurotransmitter changes may be consequent to, or parallel to Aβ peptide and phosphorylated tau accumulation in the retina.

## Introduction

Elevated levels of amyloid beta (Aβ) proteins and the associated cell damage have been described in the aging eye and opened up the possibility of Aβ being a key constituent of age-related ocular pathologies (Hinton et al., 1986; Dutescu et al., 2009; Koronyo-Hamaoui et al., 2011; Koronyo et al., 2017). Several investigations using the *Octodon degus* (degus) have confirmed a role for Aβ peptides in diseases, including increased Aβ peptides and phosphorylated tau levels in the adult retina (Acosta et al., 2014; Du et al., 2015) or altered retinal structure with age (Szabadfi et al., 2015). Other studies in degus have described behavioral and memory deficit in the animals (Inestrosa et al., 2005; van Groen et al., 2011; Ardiles et al., 2012; Castro-Fuentes and Socas-Pérez, 2013; Tarragon et al., 2013), and modeled the expression of Alzheimer’s disease–like (AD) proteins in aging degus (Inestrosa et al., 2005; van Groen et al., 2011; Ardiles et al., 2012, 2013; Tarragon et al., 2013; Inestrosa et al., 2015; Salazar et al., 2016). The degus offer new opportunities to investigate the fundamental question of whether Aβ and tau in the retina are associated with an accelerated aging process. It should also be acknowledged that although the degus have been described as a sporadic AD model with AD-like neuropathology, the occurrence of AD may be variable and not all colonies develop neuropathology in the brain and retina (Steffen et al., 2016; Bourdenx et al., 2017). As an animal model of aging or for AD investigation, it is important to consider the animal colony husbandry that includes exercise, and nutrition as these are important factors that may affect amyloidosis (Van der Auwera et al., 2005; Nichol et al., 2008; Zhao et al., 2015; Prado Lima et al., 2018). Other possible explanations may be genetic polymorphism in the degus that causes variation in enzymes which may in turn have implications on metabolic and neurological functions (Carter et al., 1972; Gurney et al., 1986; Quan et al., 2009). Inbreeding of laboratory-bred degus may also display different co-morbidities and exposure to stress compared to wild-captured bred degus (Palacios and Lee, 2013; Ibanez et al., 2018). Therefore, we conducted this investigation using tissues from a colony of degus that had occurrence of Aβ peptides and phosphorylated tau in adult animals, and animals that had behavioral testing to infer their neurological status.

In a previous publication (Du et al., 2015), greater Aβ peptide levels were observed in the retina of adult degus (average age of 28 months) than the young animals (average age of 12 months). The protein levels were also higher in central retina than in peripheral retina. Furthermore, there was an age-related increase in Aβ oligomers and hyper-phosphorylated tau accumulation, particularly in the ganglion cell layer (GCL) of the retina. The confirmed accumulation of Aβ peptide in the retina motivated this investigation of retinal structural changes, synaptic remodeling, and neurotransmitters profile as a function of aging.

A prominent feature of the aging process in the retina and brain is inflammation (Nilson et al., 2017). In this study, we investigated whether there is resident microglial response in the aging degus retina. We also hypothesized that glutamate, being the main neurotransmitter in the vertebrate retina could be altered due to the elevated levels of Aβ peptides possibly affecting synaptic function. This study employed animals from a colony of degus where the increase in Aβ peptides during the aging process has been described (Du et al., 2015).

## Materials And Methods

### Breeding Conditions and Tissue Collection

Degus were born and raised in the animal facility at Universidad de Valparaiso, Chile. Animals were bred under a 12:12 light/dark cycle in a controlled temperature environment (23 ± 1°C) with water and food *ad libitum*. Young and adult degus were housed in groups of 2–4 animals in metal cages with a chip wood-based bedding floor. Food consisted of commercial rabbit pellet, hay cubes or Prolab rat diet (RMH 3000 LABDIET). Water *ad libitum* was provided. No material for nest or social enrichment was provided to adults. Animal ages were: juvenile 4–10 months (*n* = 25), young 12–24 months (*n* = 8), adult 36–56 months (*n* = 5) and aged adult ≥ 60 months old (*n* = 15). Only animals that had behavioral testing to infer their neurological status were included in this manuscript. Animals with cataracts, usually developing in degus secondary to diabetes, were not included in this study. Representative images of the brain Aβ labeling in some animals is shown in Supplementary Figure S1.

*Octodon degus* at various ages were euthanized by intraperitoneal pentobarbital overdose injection. All experiments were in accordance with the bioethics regulation of the Chilean Research Council (CONICYT) and in accordance with the Association for Research in Vision and Ophthalmology statement for the use of animals in ophthalmic and vision research. The University of Auckland Animal Ethics Committee (permit number: AEC 001138) also approved the collection of tissues for these experiments.

### Fluorescence Immunohistochemistry

The eyes were dissected from the orbit, and fixed in 4% paraformaldehyde (PFA), 0.01% glutaraldehyde in 0.1 M phosphate buffer saline (PBS) for 30 min followed by several 10 min PBS washes. After fixation the tissues were submerged in sucrose solutions for cryoprotection and then were embedded in optimum cryosectioning medium (OCT) and were sectioned at 12 μm and collected on Superfrost Plus glass slides. Sections were maintained frozen at −20°C until immunohistochemistry proceeded. All tissues were processed under the same conditions. Retinal sections were incubated in a blocking solution containing 6% donkey or goat serum, 1% bovine serum albumin (BSA), and 0.5% Triton X-100 for 1 h, and then incubated in primary antibody (Table 1) diluted in a 3% donkey or goat serum, 1% albumin serum bovine (BSA), and 0.5% Triton X-100 solution overnight. On day 2, the retinal tissues were washed and incubated in secondary antibody solution (goat anti-mouse or goat anti-rabbit conjugated with A594 or A488; 1:500, Molecular Probes, United States) for 3 h at room temperature in the dark before thorough washes in 0.1M PBS. 4′,6-diamidino-2-phenylindole (DAPI) was added at this stage. Sections were mounted using anti-fading reagent (Citifluor, Electron Microscopy Sciences, United States) and coverslipped.

**TABLE 1:** List of primary antibodies used for immunohistochemistry and in Western blotting.

**Name**	**Host**	**Isotype**	**Epitope/Immunogen**	**Specificity**	**Dilution**	**Supplier**	**References**
PSD95	Ms	IgG2	Recombinant rat PSD95	Glutamatergic synapses in OPL and IPL of retina	1:1000	Clone 7E3-1B8 Millipore CP35	Naisbitt et al., 1999
Synaptophysin	Ms	IgG1	Presynaptic vesicles of cerebral/spinal neurons	Presynaptic vesicles in OPL and IPL of retina	1:200	Chemicon MAB5258	Rodriguez-de la Rosa et al., 2012
Choline acetyltransferase ChAT	Rb	IgG	Cholinergic neurons in brain and central nervous system	Cholinergic amacrine cells in the retina	1:100	AB143	Fox et al., 2011
Iba-1	Rb	IgG	C terminal of Iba-1 protein	Retinal microglia	1:500	Wako 019−19741	Kanazawa et al., 2002
Glutamate	Rb	IgG	Small Molecule conjugated to BSA by a Glutaraldehyde linker	Calibrated against a spectrum of antigens to assure hapten selectivity. No measurable cross-reactivity	1:500	Abcam ab9440	Kalloniatis et al., 1996; de Souza et al., 2012
Glutamine	Rb	IgG	Small Molecule conjugated to BSA by a Glutaraldehyde linker	Calibrated against a spectrum of antigens to assure hapten selectivity. No measurable cross-reactivity (<1:1000) was detected against glutamine in peptides or proteins	1:1000	Abcam ab9445	Kalloniatis et al., 1996
GABA	Rb	IgG	Small Molecule conjugated to BSA by a Glutaraldehyde linker	Fixed tissue cross-reactivity tested with known targets at recommended dilution. No measurable glutaraldehyde-fixed tissue cross-reactivity (<1:1000) against other amino acids	1:500	Abcam ab9446	Kalloniatis et al., 1996; de Souza et al., 2012
Pan-Shank	Ms	IgG1	Recombinant protein consisting of SH3/PDZ domain of rat Shank2	Western Blotting in rat brain membrane tissue lysate	1:500	Clone N23B/49; UC Davis/NIH NeuroMab	Serrano et al., 2014
GluA2	Ms	IgG1	Fusion protein amino acids 834–883 cytoplasmic C-terminus of rat GluA2/GluR2	Immunoblot of membranes from adult rat brain and adult GluA2/GluR2 knockout and wild-type mouse hippocampi	1:1000	Clone L21/32; UC Davis/NIH NeuroMab	Serrano et al., 2014
GluN1	Ms	IgG2	Recombinant protein corresponding to extracellular N-terminus of rat GluN1/NR1	Specific for GluN1	1:1000	Clone N308/48 UC Davis/NIH NeuroMab	Uttl et al., 2018

### Silver Intensified Immunogold Labeling

The procedure for post-embedding silver-intensified immunogold detection has been previously described (Marc et al., 1990; Acosta and Kalloniatis, 2005; Sun et al., 2007). Briefly, eyes were dissected out of the orbit and fixed in 2.5% glutaraldehyde, 1% PFA in 0.1 M PBS for 1 h followed by several 10 min PBS washes. The retina was then dissected out of the eye and was processed for embedding in Epon resin. The tissue was sectioned at 500 μm thickness using a Leica Ultracut UCT ultramicrotome (Leica, Germany). Retinal sections were collected on PTFE (Teflon)-coated 10-well slides (ER-208B-CE24, Thermo Fisher Scientific) and stored until immunohistochemistry proceeded.

Primary antibodies used in this procedure detected retinal amino acids (Table 1: anti-glutamate; anti-glutamine; anti- gamma-aminobutyric acid), and 1.4 nm Nanogold^®^ conjugated secondary antibody (1:100; #2003, Nanoprobes, United States) was used to detect the primary antibody. The detection was enhanced using silver intensification of the nanogold tag as previously described (Kalloniatis et al., 1996).

### Western Blotting

Retinal samples were processed as described in a previous publication (Du et al., 2015). Briefly, proteins extracted from homogenized retinas were resolved by 10% SDS-PAGE and transferred to a polyvinylidene difluoride (PVDF) membrane. Proteins of interest were detected using primary antibodies raised in mouse (Table 1) and a secondary goat anti-mouse peroxidase conjugated antibody (Pierce, United States). Band intensities in Western blotting were visualized with enhanced chemiluminescence blotting substrate (Pierce, United States) and densitometrical quantification was conducted using Image J software (National Institutes of Health, United States). Data are presented as relative values to the loading control protein value.

### Tissue Imaging

All fluorescence retinal images were acquired using an Olympus FluoView^TM^FV-1000 confocal microscope (Olympus Corporation, United States), with the excitation wavelengths set for Alexa 488, and Alexa 594 fluorochromes and for DAPI. A z-stack (along the optical axis) was acquired using 0.9 μm step-size to go through a total thickness of ∼9 μm per tissue. Six retinal images acquired from different animals were analyzed for each group, and the representative images shown in the figures. Retinal sections with silver-intensified amino acid immunoreactivity were imaged under the same lighting and contrast parameters, using Brightfield microscope (Leica Corporation, Germany) with an attached Leica DFC495 camera and a LASV4.8 Leica Microsystems software used for taking the images.

### Quantification of Labeling

Six images from each animal per age group were analyzed to obtain the percentage area labeled by the antibody, using Image J (National Institutes of Health, United States). Images were split into RGB channels and the single channel that corresponded to the secondary antibody fluorochrome was used in the quantification. Retinal cell counting was performed in 1 μm thickness confocal images. A fixed size area was selected on each image and the number of labeled cells in that area was counted. Number of Iba-1 positive microglial cells were counted in central retina. Values per retina were averaged and plotted as mean ± SEM (standard error of the mean).

Immunogold labeling was quantified in grayscale images. Using the auto-threshold modality, minimum and maximum threshold values were selected. The labeled area was calculated by determining the mean pixel number in selected retinal areas.

### Statistical Analysis

Statistical analyses were performed using SPSS (International Business Machines Corporation, United States). One-way ANOVA and Bonferroni *post hoc* test was performed to determine significant differences in the expression of retinal markers between age groups. A *p* < 0.05 was considered to be statistically significant.

## Results

### Evidence of Retinal Synaptic Remodeling as a Function of Aging in Degus

**FIGURE 1:**
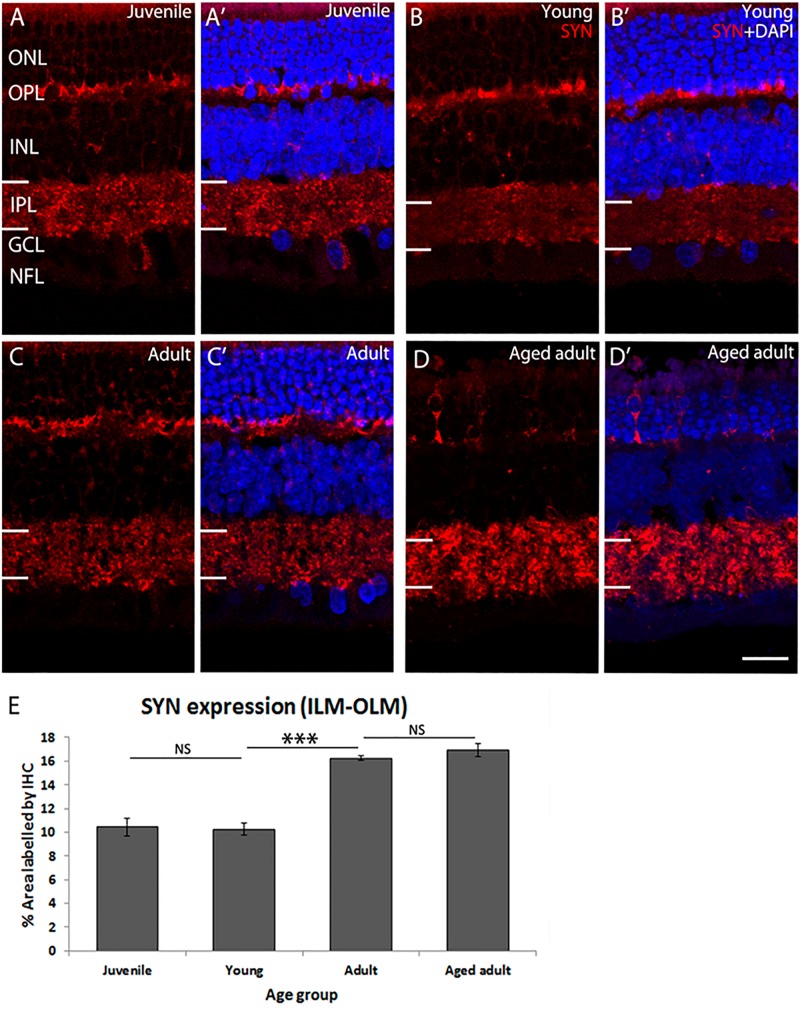
SYN immunolabeling in degus retina. **(A,A’)** Juvenile degus, **(B,B’)** Young degus, **(C,C’)** adult degus, **(D,D’)** aged adult degus. **(E)** Quantification of percentage area occupied by SYN labeling in the entire retinal image. SYN, synaptophysin; OLM, outer limiting membrane; ONL, outer nuclear layer; OPL, outer plexiform layer; ILM, inner limiting membrane; INL, inner nuclear layer; IPL, inner plexiform layer; GCL, ganglion cell layer; NFL, nerve fiber layer. Scale bar = 20 μm. Statistical analysis was completed by one way ANOVA. Data are expressed as mean ± SEM (*n* = 6). Significant values are indicated with asterisks: ^∗∗∗^*p* < 0.001.

The retina was immunolabeled to confirm accumulation of Aβ peptides and Tau phosphorylation (Supplementary Figures S2, S3). In these tissues, we have previously determined that there are changes in retinal ganglion cell (RGC) density and photoreceptor cell apoptosis in adult retina (Du et al., 2015). Now we investigated whether the retinal plexiform layers are affected. Synaptophysin (SYN) was detected in presynaptic structures in the outer plexiform layer (OPL) and inner plexiform layer (IPL) of the degus’ retina (Figure 1). SYN expression was similar in juvenile and young retina (juvenile: 10.44 ± 0.72 vs. young: 10.27 ± 0.52, *p* = 0.996). However, there was a marked increase in SYN expression in adult degus (adult: 16.26 ± 0.2, *p* < 0.001). There was no statistically significant difference between adult and the aged adult group (16.94 ± 0.57, *p* = 0.809). However, SYN labeling pattern showed retraction of photoreceptor synaptic end (Figures 1D,D’, arrows), and reduced labeling of the OPL.

**FIGURE 2:**
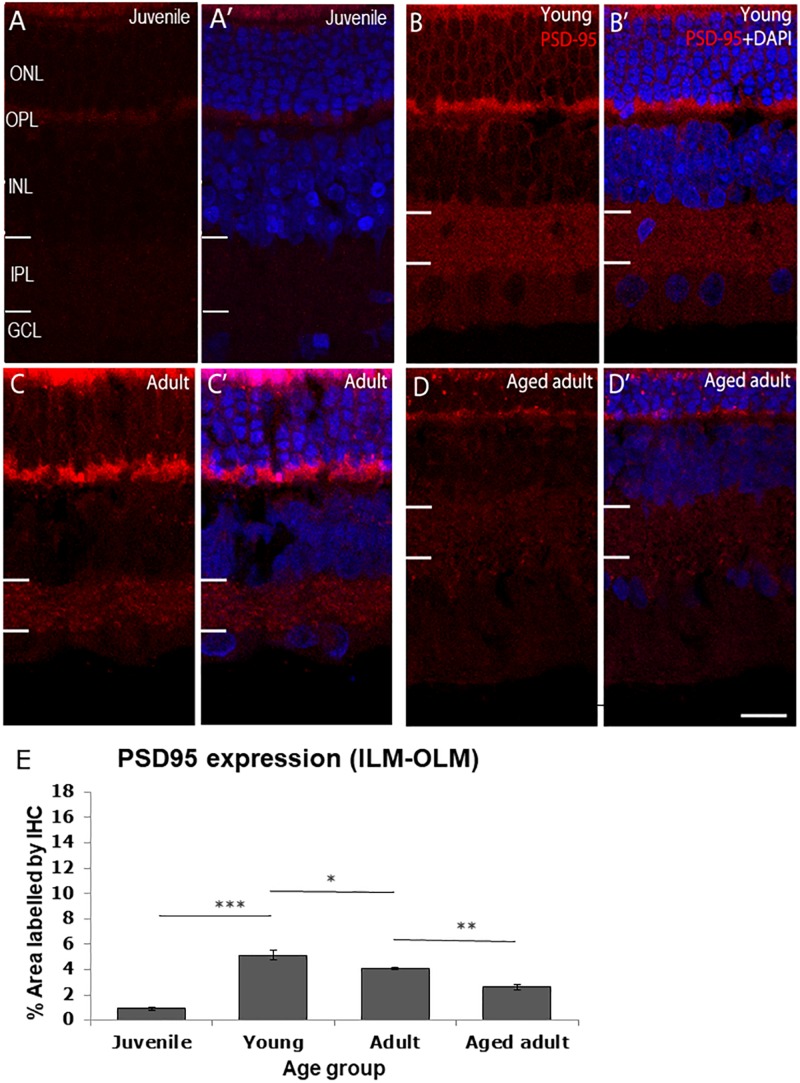
PSD95 immunolabeling in the degus retina. **(A,A’)** Juvenile degus, **(B,B’)** Young degus, **(C,C’)** adult degus, **(D,D’)** aged adults. **(E)** Quantification of % area occupied by PSD95 labeling in the entire retinal image. Intense labeling in the outer segment seen in **(C,C’)** is judged to be unspecific autofluorescence. PSD95, post-synaptic density protein 95; OLM, outer limiting membrane; ONL, outer nuclear layer; OPL, outer plexiform layer; ILM, inner limiting membrane; INL, inner nuclear layer; IPL, inner plexiform layer; GCL, ganglion cell layer; NFL, nerve fiber layer. Scale bar = 20 μm. Statistical analysis was completed by one way ANOVA. Data are expressed as mean ± SEM (*n* = 6). Significant values are indicated with asterisks: *p < 0.05; **p < 0.01; ***p < 0.001.

The post-synaptic density protein 95 (PSD-95) was detected in the OPL and IPL (Figure 2). PSD-95 expression was the lowest in juvenile degus in which < 1% of the retina was immunolabeled. There was a statistically significant increase in PSD-95 protein expression from juvenile to young age (juvenile: 0.91 ± 0.15 vs. young: 5.12 ± 0.4, *p* < 0.001), which remained high in adults (4.07 ± 0.8) but was significantly different from young animals (*p* < 0.05). Interestingly, a significant decrease in protein expression was found in aged adults (2.60 ± 0.22) compared with adult animals (*p* < 0.01). The decrease in PSD-95 protein expression was found even in aged adults that had a reduction in size of both the outer nuclear layer (ONL) and the OPL (Figures 2D,D’).

### Quantification of Synaptic Proteins and Glutamate Receptor Subunits

**FIGURE 3:**
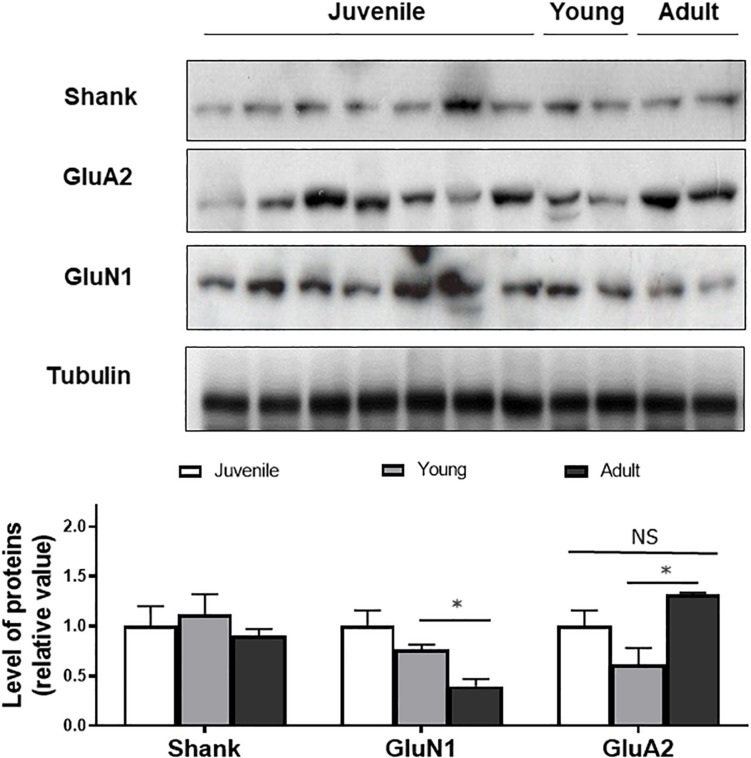
Western blot of Shank, GluN1, GluA2 in juvenile, young, and adult retina. Quantification of the bands shows no age-related changes in expression of the markers, except for GluN1. Anti-tubulin was used as loading control. Statistical analysis was done using by one way ANOVA with Boferroni *post hoc* test. Data are expressed as mean ± SEM (*n* = 6). Significant values are indicated with asterisks: ^∗^*p* < 0.05; NS, non-significant.

To determine if the changes in synaptic layer protein expression were associated with neurotransmitter changes, Western blotting of synaptic proteins and glutamate receptor subunits was conducted (Figure 3). Shank was used as a post-synaptic density protein marker which functions as a part of the N-methyl-D-aspartate (NMDA) receptor-associated PSD-95 complex (Naisbitt et al., 1999). It has both signaling and anchoring functions. Shank expression was found to be relatively constant as a function of age.

GluN1, a subunit present in NMDA receptors was also quantified as a function of degus’ age. GluN1 expression was not significantly different between juvenile and young animals but a significant decrease was detected between young and adults (*p* < 0.05), suggesting an age-related effect on expression of the subunit.

GluA2, a subunit of post-synaptic α-amino-3-hydroxy-5-methyl-4-isoxazolepropionic acid (AMPA) type glutamate receptors was also found at all ages. There was a non-significant decrease in GluA2 expression when comparing juvenile with young retina. A significantly higher expression was seen in the adult degus group (1.31 ± 0.02) compared with the young group (0.61 ± 0.05), but the GluA2 value in the adult group was not significantly different from the juvenile group (*p* = 0.34; Figure 3).

### Aging in Degus and Neurotransmitter Levels

**FIGURE 4:**
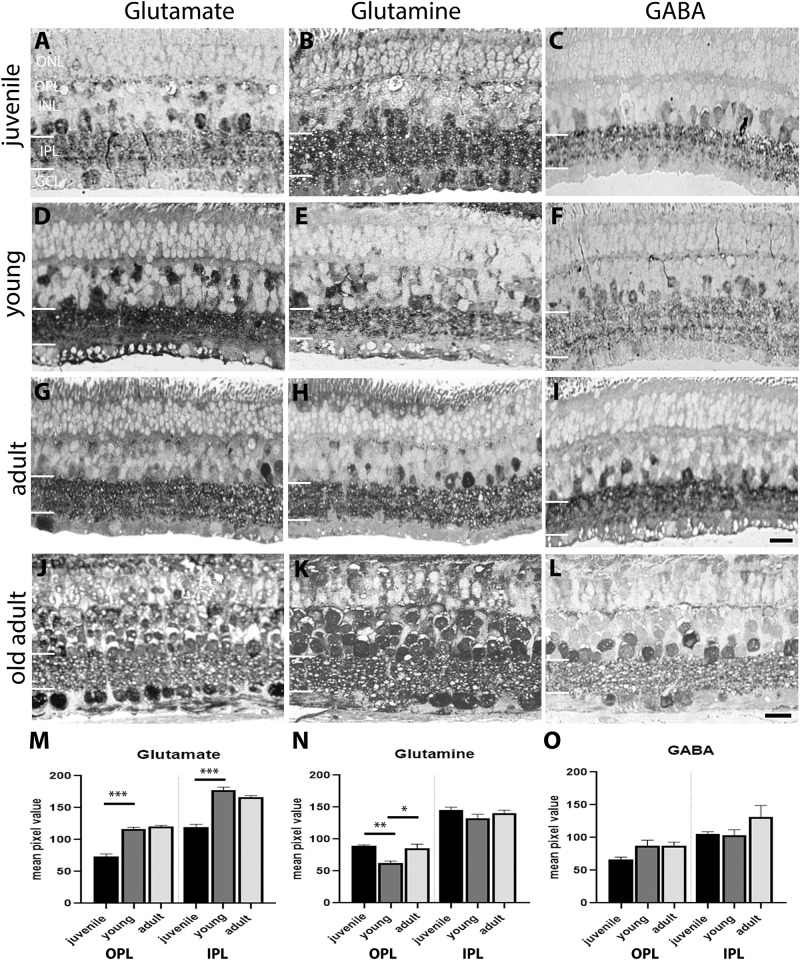
Aminoacid immunolabeling in the degus retina. **(A,D,G,J)** Glutamate, **(B,E,H,K)** Glutamine, **(C,F,I,L)** gamma-aminobutyric acid (GABA) Glutamate labeling was most different in juvenile retina **(A)** compared with young **(D)** and adults **(G)** where there was labeling throughout the retina. Glutamine in juvenile degus **(B)** was observed in the IPL and INL, while in young retina **(E)** glutamine expression was increased in areas corresponding to the location of Müller cells. High level of glutamine immunolabeling was seen in amacrine cells in the adult retina **(H)**. GABA in juvenile **(C)** and in the young retina **(F)** was expressed in amacrine cells in the INL and immunolabeled sublayers in the IPL, while in adults **(I)** labeling increased in the INL and extended to labeling of the OPL. In the old adult, increased expression of glutamate **(J)**, glutamine **(K)**, and GABA **(L)** was observed. Quantification of the labeling in juvenile-adult ages is shown in **(M–O)**. There were not enough silver intensified immunogold samples to quantify the old adult group. Scale bar on **(I)** = 20 μm and applies to **(A–I)**. Scale bar on **(L)** = 20 μm and applies to **(K,L)**. Statistical analysis was completed by one way ANOVA. Data are expressed as mean ± SEM (*n* = 4–5). Significant values are indicated with asterisks: ^∗^*p* < 0.05; ^∗∗^*p* < 0.01; ^∗∗∗^*p* < 0.001.

The observed changes in synaptic protein expression and in glutamate receptor subunit prompted us to investigate the levels of glutamate neurotransmitter, and other amino acids related to the glutamate pathway (Figure 4). An anti-glutamate antibody was used to immunolabel degus’ retina. In juvenile retina, glutamate was expressed in the IPL, in some bipolar cells (BC), amacrine cells (AC), and in the GCL. Young retinas showed increased expression in the IPL, AC, horizontal cells (HC), BC, and the GCL. Quantification of the amount of labeling in the plexiform layers showed an increase in glutamate in the IPL and OPL at young age (Figures 4M,N and Supplementary Table S1). Such increase was maintained in adult retina (Figure 4J).

Glutamine was expressed in all layers but more abundantly in the INL and in the IPL in juvenile degus. The expression pattern was similar in the young group, but Müller cell processes were clearly labeled. Quantification of the amount of glutamine labeling in the OPL showed a significant decrease (*p* < 0.01) followed by increase (*p* < 0.05) in the OPL of the old adult degus (Figure 4K). No changes were observed in the IPL compared with adults.

GABA immunoreactivity was assessed as a function of age. Strong labeling was found in AC, and three typical GABA-positive bands were present in the IPL in juvenile retina (Figure 4C). The labeling pattern was similar in juveniles and in young (Figures 4C,F). Some areas in the OPL and Müller cell processed in the ONL were immunoreactive to GABA. The adult retina showed a similar pattern of GABA with no significant changes in the plexiform layers (Figure 4I). Such GABA expression pattern and labeling in adult retina has been described in other species (Kalloniatis and Tomisich, 1999). The labeling pattern did not appear to differ from the old adult age group (Figure 4L).

**FIGURE 5:**
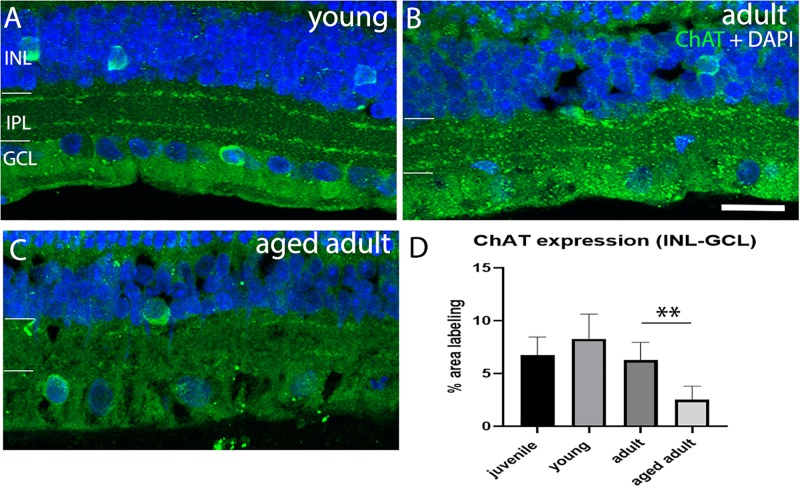
Choline acetyltransferase immunolabeling in degus retinas. **(A)** Young degus, **(B)** adult degus, **(C)** aged adult degus. ChAT labeling was similar in young retina compared with juvenile. Adults had reduced labeling of the IPL and apparent reduction in labeling of somata in the INL and GCL. Aged adults had a reduction in labeling of the IPL but intense ChAT labeling was seen in the few remaining labeled cells. **(D)** Quantification of ChAT expression per unit area was conducted in *n* = 4 retina per group. Statistical analysis was completed by one way ANOVA. Significant values are indicated with asterisks: ^∗∗^*p* < 0.01. Scale bar = 20 μm.

Loss of cholinergic neurons has been reported in AD in the brain. We investigated whether cholinergic AC were affected in the degus model as a function of age. The results show that a juvenile and young retina show a normal pattern of ChAT^+^ cell arrangement in the INL and in the GCL with strong labeling of two sublayers in the IPL (Figure 5A). Adult retinas also show this typical arrangement in the IPL, but less labeling of somata was observed in the nuclear layers (Figure 5B). Aged adult degus, on the other hand, presented a decreased number of ChAT^+^ somata and highly reduced labeling of the IPL (Figure 5C). Quantification of the labeling confirmed reduced ChAT expression in the aged adult degus (Figure 5D).

### Microglial Phenotype in the Retina

**FIGURE 6:**
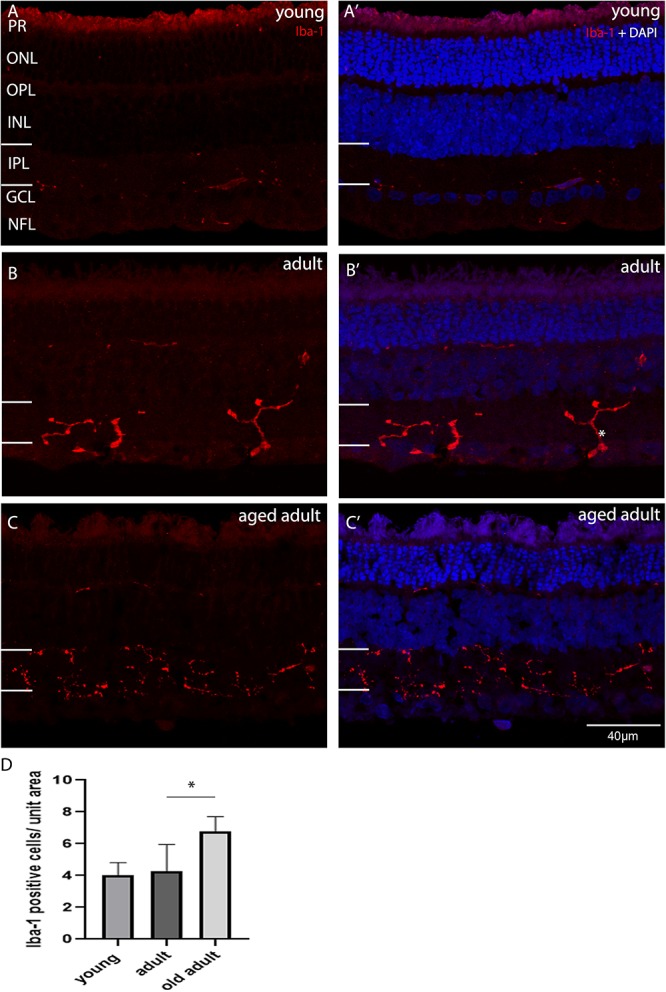
Iba-1 immunolabelling of degus central retina. **(A’)** Young, 18-month old degus, **(B’)** Adult, 48-month old degus, **(C’)** Aged adult, 84-month old degus. Representative images of increased activation of microglia as a function of age. **(D)** Quantification of number of Iba-1 per unit area was conducted in *n* = 4 retina per age group. Young retinas had inactive microglial cells **(A)**, while they were dendritic-like in adult retina. **(B)**. The aged adult group did not seem to have active microglia **(C)**. Statistical analysis was completed by one way ANOVA. Significant values are indicated with asterisks: *p < 0.05. Scale bar = 40 μm.

In the retina, microglia have a role in neuroprotection and degeneration. The role is evident when there is change in the morphology of resident microglia. Microglia was considered to be inactive, intermediate activation or active according to the morphological description published by Karperien et al. (2013). Unramified and intermediate forms were considered to be active vigilant sentinels. Amoeboid or rounded microglia were considered to be active with an inflammatory role. In their unramified and intermediate forms microglia are considered to be activated (Karperien et al., 2013). In their fully hyper-ramified form microglia are actively engaged in essential physiological roles, such as those described in Karperien et al. (2013). Microglia in its intermediate morphological forms may be associated with synaptic remodeling, as a reduction in PSD95 expression, and increase in SYN expression was seen when animals transitioned from young to adult age. To determine whether the degus retina was in an active or intermediate phase of surveillance, as the result of abnormal accumulation of Aβ peptides, the synaptic remodeling and the neurotransmitter changes, microglia was labeled with ionized calcium-binding adaptor molecule-1 (Iba-1) and its morphology assessed as a function of age. Iba-1 labels resting and active microglial cells and we classified the cells according to their morphology. Figure 6 shows the Iba-1 positive cells in central retina in each age group. Figures 6A,A’ show that the microglial cells were mostly inactive, as they were flat and had short unramified processes within their usual residing retinal layer in juvenile and young degus retina. Microglial cells in the IPL were more of a “dendritic-like” appearance (Rojas et al., 2014), had longer cell processes projecting beyond the IPL, and were found in young retina but mostly in adult (Figure 6B). The aged adult group had more microglia per unit area in central retina (Figures 6C,D). However, their morphology did not reflect an active stage of inflammation. A particular microglia morphology of intermediate activation was first noticed at ages between 12 and 48 months old, and tissues within this age-bracket were further analyzed.

**FIGURE 7:**
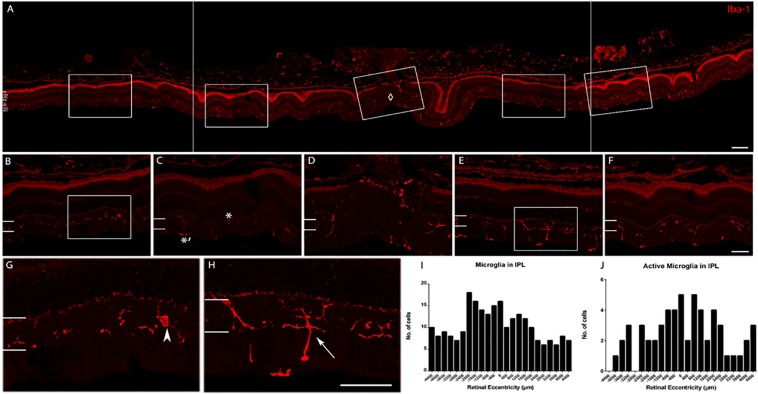
Iba-1 immunolabeling of a retina representative of the young age group. **(A)** Collage of retinal images spanning ∼2200 μm on either side of the optic nerve head (ONH, marked by ♢). Note the high labeling of Iba-1^+^ cells in the inner retina and the choroid. **(B)** Enlarged view of the peripheral retina (∼1952 μm to the left of ONH). **(C)** Enlarged view of the central retina (∼1120 μm to the left of ONH. Active microglial in IPL is indicated with ^∗^ and inactive microglia with ^∗^′ in the NFL. **(D)** Enlarged view of the ONH area. **(E)** Enlarged view of the central retina (∼672–1040 μm to the right of ONH). **(F)** Enlarged view of the peripheral retina (∼1200–1552 μm to the right of ONH). **(G)** Magnified view of box in **(B)** (arrowhead: microglia cell in the peripheral retina). **(H)** Magnified view of box in **(E)** (arrow: active cell in the central retina). **(I)** Quantification of microglial cell count in the IPL. **(J)** Quantification of active microglial cells in the IPL. Scale bar = 80 μm in **(A)**, 40 μm in **(B–H)**.

We determined whether the microglia morphology was consistent across retinal eccentricity in the young age animals. A detailed analysis of the labeling pattern as a function of eccentricity was conducted, and a representative image shows labeling around the optic nerve head (ONH) in Figure 7. In the young retina, there were active and inactive microglia as a function of eccentricity. The morphology of the cells varied depending on the distance from the ONH, and probably with retinal position. Quantification of the number of cells as a function of distance from the optic nerve showed that active microglial cells were within an area spanning ∼1200 μm on either side of the optic nerve. The active state of microglia was judged by the number of hyper-ramified processes (up to four fine and elongated cell processes) branching out. Iba-1 cells that were more than 1200 μm away from the ONH have less, thicker, and shorter cell processes (inactive).

## Discussion

**FIGURE 8:**
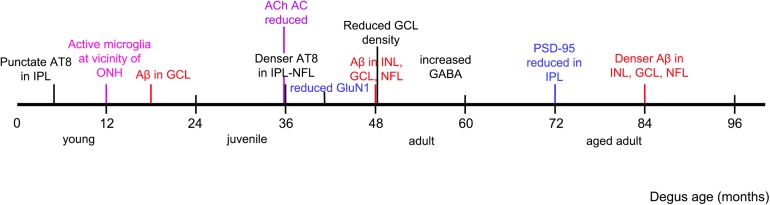
Observed chronology of protein and synaptic changes in degus. Timeline is marked as per the youngest age of degus investigated that had an observed phenomenon, such as accumulation of specified protein, and structural changes in the retina.

This investigation confirmed the presence of synaptic changes with aging in degus retina. We proposed that synaptic remodeling is the consequence of abnormal accumulation of Aβ peptides and hyperphosphorylated tau, found in degus at a young age (∼12 months old) (Du et al., 2015). This is in agreement with Masliah and colleagues’ (Masliah et al., 2006) suggestion that the mechanism of synaptic loss is due to excess Aβ accumulation reaching neurotoxic levels, resulting in dysregulation of glutamate levels at synaptic clefts, and neurite degeneration. In addition, we have identified in young degus microglial intermediate activation near the ONH, with decreasing activity toward the peripheral retina, suggesting that the reported accumulation of the AD-related proteins in central retina (Du et al., 2015) elicits microglial activation for maintenance of synaptic structures (Rashid et al., 2019). Analysis of tissues at selected ages allowed us to determine that there is a clear sequence of events in protein expression. This is summarized in Figure 8.

### Age-Related Decrease of Cholinergic Amacrine Cells Occurs After Aβ Accumulation and Coincides With the Age When Dense Phosphorylated Tau Accumulates in IPL-NFL

In the AD-like brain, altered neuritic growth and synapse formation are associated with abnormal processing of amyloid precursor protein (APP) and Aβ aggregation. This results in degeneration of sprouting cholinergic neurites into dystrophic forms similar to what is observed in mature AD plaques. Zheng et al. (2002) also reported an increase in tau phosphorylation and loss of cholinergic neurons that were time and Aβ-concentration dependent in rat primary septal cultures (Zheng et al., 2002). This is in accordance with the amyloid hypothesis that abnormal Aβ accumulation is the upstream event that initiates cascading events and neuroinflammation (Hardy and Selkoe, 2002). What we observed in degus’ retina appears to resemble an intermediate activation process seen in the brain, as retinal cholinergic AC had an age-related decrease in number and synapses in the IPL. Cholinergic AC are also known as starburst AC. They synapse in both the ON and OFF sublaminae of the IPL with directionally-selective RGC that are responsive to image motion. Deficits in global motion and dynamic visual tasks have been described in the literature, but were often attributed to degenerative process in the visual processing areas of the AD brain (Rizzo and Nawrot, 1998; Vallejo et al., 2016). Further investigation is warranted to address whether such functional deficit in AD patients may also be due to reduced cholinergic synaptic input to directionally-selective RGC.

### Age-Related Change of Post-synaptic Marker Expression Is a Later Event Than Aβ Accumulation and Microglia Activation

There was differential PSD-95 expression with age in the degu’s retina. PSD95 was the least abundant in the juvenile degus, and peaked in the young degus, followed by a decrease in protein expression from adult age. PSD is a complex network of neurotransmitter receptors, regulators of synaptic electrical activity, and links to cytoskeletal elements. PSD95 has been previously described to strongly label the rod spherules and cone pedicles in the OPL. It is also found at BC ribbon synapses in the IPL, and co-localizes with the NR1 subunit of the NMDA glutamate receptors (Koulen et al., 1998). The low PSD95 expression in the juvenile degus may be due to a mechanism similar to the repression during neural development as described by Zheng et al. (2002) in the mice embryonic brain (Zheng et al., 2002). PSD95 RNA was successfully transcribed in these mice, but had unsuccessful translation into proteins, which led to degradation. We speculate that as the degus matured from juvenile to young age, PSD95 formation became more normalized and reached the normal physiological level. In fact, the scaffolding arrangement with Shank was not altered. On the other hand, the reduction of PSD95 from adult age may be due to aging and amyloidosis. This theory is supported by the study by Yuki et al. (2014) in which AD donor brain regions had marked Aβ deposition and reduced level of PSD95, post-synaptic disruption and neuronal loss (Yuki et al., 2014). Similarly, Ardiles et al. studied the degus brain by immunoblot, and found PSD95 levels to be significantly reduced in animals 36 months of age (Ardiles et al., 2012), which corresponds to adult age in this current study. However, a difference in PSD95 expression was not seen from juvenile to young age in the degus brain. An interesting observation was the prominent reduction in PSD95 expression in the IPL, which coincidentally was the location of intense paired helical filaments (PHF)-tau labeling in adult degus (Du et al., 2015). This may imply that PHF-tau accumulation is particularly detrimental for the post-synaptic function in retinal neurons, as it is in the brain (Avila et al., 2004).

SYN is a membrane protein of the synaptic vesicles, and has multiple functions in synaptic vesicle formation, exocytosis and delivery of neurotransmitters. It is also thought to be closely related to synaptogenesis and synaptic plasticity during neural tissue development (Dan et al., 2008; Yuki et al., 2014). In the human AD donor brain SYN protein level was decreased but not in control normal tissues. This suggested that presynaptic disruption, as well as post-synaptic disruption is consequent to abnormal protein deposition in AD. It is unclear why an increased SYN level, rather than reduced was found in the adult/aged adult degus retina that were more likely to be affected by Aβ aggregation. One possible explanation may be that although the retina and the brain are both neuronal tissues they may have different response mechanisms to pathology. In a study by Dan et al. (2008) SYN expression was found to be upregulated in the rat retina following an acutely induced high intraocular pressure, both in its mRNA and protein form, and that the distribution of SYN was broadened (Dan et al., 2008). These results suggested that aside from degeneration in events of cellular stress, retinal synapses may also undergo regenerative events that involve regulation of neurotransmission. We observed that the expression pattern of glutamate in degus was comparable with the expression in retinal neurons across species (Kalloniatis et al., 1996). However, the labeling observed in Müller cells was abnormal, and as shown in other species, redistribution of neurotransmitters to these cells indicates that there is stress in the retina (Acosta et al., 2005). In this study, glutamate and glutamine labeling pattern signaled neurochemical remodeling starting at a juvenile age in degus. Other studies have suggested that Aβ oligomers (present at a young age in the retina of degus) reduce glutamatergic synaptic transmission by decreasing of AMPA and NMDA receptors expression (Shankar et al., 2007). In adults, the labeling pattern was not different but as seen in other chronic models of retinal degeneration, a shift toward increased excitability is observed. Our findings in the degus, together with the interesting data presented by Antes et al. (2013) on apolipoprotein E4 (apoE4) targeted replacement mice (a transgenic model with apoE4 being the most prevalent genetic risk factor for AD) arrive to similar conclusions that changes at presynaptic terminals and glutamatergic nerve terminals may be preferentially affected (Antes et al., 2013). This motivates further study in the synaptic activities in the degus retina.

### Intermediate Stages of Microglial Activation in the Retina Indicates Synaptic Remodeling Starts at a Young Age

Microglial cells showed morphological intermediate forms of activation through the retina at an adult age (≥36–48 months), which coincided with the same age groups that have Aβ and Tau protein changes, and showed the greatest increase in previous investigations (Du et al., 2015; Grimaldi et al., 2018). One of the key observations in the microgliosis of the degus’ retina was that activated microglia were significantly more numerous in the central retina (closer to the ONH) than the peripheral retina. This is explained by the degus’ retina anatomy, in which the RGC density is the densest in the visual streak area that is proximal to the ONH. The degus RGCs have also been studied and there are ∼300,000 cells with varying density at different retinal eccentricity (Vega-Zuniga et al., 2013). Although there is no discernible macula in rodents, there is a circumscribed and well-developed *area centralis* region ∼2.8 mm dorso-temporal to the ONH and measures about 1.0 mm^2^. In the *area centralis* the peak RGC density is 6,384 cells/mm^2^ (Vega-Zuniga et al., 2013). The *area centralis* is located within the visual streak, where the visual acuity is the highest – it runs nasotemporally above the ONH. As the Aβ and NFT were both found to accumulate in the inner retina (NFL, GCL, and IPL) of the degus, and these layers are the axonal, nuclear, and dendritic processes of the GCL respectively, it is possible that active microglia were the most numerous in the central retina due to relatively greater density of RGCs, and greater amount of Aβ and NFT accumulation. Very few completely amoeboid cells were observed, suggesting that the increased number of microglia around the ONH may be associated with local changes rather than an active state of inflammation. It was also interesting to note that the microglia in the IPL seemed intermediately activated over the microglia in the OPL, which stayed inactive (flat cell morphology with few cell processes). This may be because the inner retina was exposed to relatively more cellular stress, possibly due to the Aβ and NFT deposition.

### Degus May Be an Accelerated Model of Age-Related Retinal Change and Neurodegeneration

Aβ accumulation in the retina is a sign of amyloidosis associated neurodegeneration (Koronyo-Hamaoui et al., 2011), and is also observed in diabetes (Bitel et al., 2012). In degus, diabetes is strongly associated with the incidence of cataracts (Ardiles et al., 2013). In this study, animals with no lens opacities were employed, allowing us to conclude that Aβ deposition is not due to diabetic comorbidity. Over the course of normal aging, Aβ has been observed as deposits in the retina of mice and humans (Hoh Kam et al., 2010). However, in neurodegenerative diseases with Aβ accumulation in the retina, such as in AD (Blanks et al., 1996a, b) peptide accumulation is seen in the retina before the brain (Chang et al., 2014, 2015; Du et al., 2015). Aβ accumulation also occurs during glaucoma and age related macular degeneration (ARMD). In ARMD, soluble Aβ, mature Aβ fibrils (Isas et al., 2010) and tau are all found in drusen deposits and lead to local toxicity of the retinal pigment epithelium (RPE) in ARMD in humans. In an investigation by Shelley et al. (2009), retinal synaptic changes were noted in human ARMD tissue, where aberrant distribution of immunoreactivity for vGluT1 was noted in the cone axon and pedicle in ARMD, while vGluT1 transporter was normally confined to the presynaptic terminal. This aberrant distribution may be the result of synaptic remodeling as described by Sullivan et al. (2007), where immunolabeling of sections of human retinas affected by ARMD showed a redistribution of SYN and vGLUT-1 from the OPL (seen in normal retina) to the ONL. Furthermore, the OPL morphology was disordered and appeared reorganized, suggesting the retraction of photoreceptor axonal processes and their synapses back into the ONL. This is also accompanied by subsequent outgrowth of dendrites from the post-synaptic bipolar cells, and reformation of synaptic contacts between photoreceptor and bipolar cells. Collectively, these findings demonstrate that aged and degenerating retinal tissues have the tendency and capacity to undergo synaptic remodeling. In light of this, it may be plausible to infer that the synaptic changes seen in the degus retina are also due to aging and underlying degenerative processes.

Degus have been described as a novel AD-like animal model (Inestrosa et al., 2005; van Groen et al., 2011; Ardiles et al., 2012, 2013; Castro-Fuentes and Socas-Pérez, 2013; Tarragon et al., 2013; Vega-Zuniga et al., 2013; Acosta et al., 2014; Du et al., 2015; Inestrosa et al., 2015; Szabadfi et al., 2015; Altimiras et al., 2017; Cisternas et al., 2018). However, investigators have also found that degus housed and bred under different conditions may result in different levels of AD-protein accumulation. Contributing factors may include enrichment effects, genetic polymorphism, and inbreeding/outbreeding (Rivera et al., 2016). Further research is warranted to investigate the extent each of the aforementioned factors play in the varied observations of AD-protein accumulation in the degus.

## Conclusion

By employing degus at different ages we demonstrated that there is an age related increase in Aβ accumulation in the retina that is associated with synaptic changes and neurotransmitter imbalance. The aged adult degus showed variability in expression of AD markers, as expected in a non-transgenic natural animal model. This reiterates that degus are a close representation of the aging process and sporadic/late-onset AD, as these animals share 97.5% protein homology with human Aβ.

## Data Availability Statement

All datasets generated for this study are included in the article/Supplementary Material.

## Ethics Statement

The animal study was reviewed and approved by bioethics regulation of the Chilean Research Council (CONICYT) and the University of Auckland Animal Ethics Committee (permit number: AEC 001138).

## Author Contributions

LC, MA, and AP conceptualized the study. LC contributed to the acquisition, analysis and interpretation of data, contributed to the conception of the work, drafted the manuscript, and revised it critically for intellectual content. AP, MA, and NI assisted with funding acquisition. AA and CT-R contributed to the acquisition of data, analysis and interpretation of data, contributed to the conception of the work, and revised the manuscript critically for intellectual content. JA contributed to the processing of tissue samples and acquisition of data. All authors contributed to writing, reviewing and editing the final version. All authors read and approved the final manuscript.

## Conflict of Interest

The authors declare that the research was conducted in the absence of any commercial or financial relationships that could be construed as a potential conflict of interest.

## Supplementary Material

FIGURE S1 Aβ4G8 immuno-labeling of the degus brain. **(A)** No labeling in the Juvenile 5 month old degus. **(A’)** Magnified view of inset in **(A)**. **(B)** positive labeling in the Aged Adult 96 month old degus (marked by ^∗^ in the hippocampus and ^∗^’ in the cortex). **(B’)** Magnified view of inset in **(B’)**. Scale bar = 500 μm for **(A,B)**, 200 μm for **(A’,B’)**.

FIGURE S2 Expression of amyloid precursor protein (APP) **(A,F,F’,K,K’P,P’)**; accumulation of amyloid β peptides detected with Aβ4G8 in **(B,G,G’L,L’,Q, Q’)**, Aβ6E10 in **(C,H,H’, M, M’,R,R’)**, Aβ11A5 in **(D,I,I’, N, N’, S, S’)** and Aβ12F4 in **(E,J,J’,O,O’)** as a function of development. **(A–E)** Juvenile degus, **(F–J)** young degus, **(K–O)** adult degus, **(P–S)** aged adult degus. APP A4 (clone 22C11) is a mouse antibody, IgG1 raised against amino acids 66–81 in the N terminus of human APP (1:100 Abacus-als, MAB348). Aβ4G8 is a mouse antibody, IgG2b raised against amino acids 17–24 of human Aβ peptide (1:300 Huntingtree SIG-39220). Aβ6E10 is a mouse antibody IgG1 against amino acids 1–16 of human Aβ peptide (1:300 Huntingtree SIG-39320). Amyloid β, clone 11A5-B10 is a mouse antibody IgG against amino acids 3–40 of human Aβ (1:100 Millipore 05-799). Amyloid β12F4 is a mouse antibody IgG1 against amino acids x-42 of human Aβpeptide (1:500 Covance SIG-39142).

FIGURE S3 PHF-Tau (AT8) immuno-labeling in the degus retina. **(A)** Juvenile degus, **(B)** young degus, **(C)** adult degus, **(D)** aged adult degus. The ^∗^ indicates punctate labeling in the IPL and arrowheads indicate cytoplasmic deposits. **(A’–D’)** Magnified view for PHF-Tau corresponding to each age inset. **(A”–D”)** Magnified view for PHF-Tau + DAPI. **(E)** Quantification of the % area occupied by PHF-Tau in total retina (ILM-OLM). **(F)** Quantification of % area occupied by PHF-Tau in the NFL-GCL in all groups. PHF-tau (clone AT8) is a mouse antibody, IgG1 raised against phosphorylated Ser202/Thr205 in human PHF-tau (1:200 Thermo Fisher MN1020). Abbreviations: PR, photoreceptors; ONL, outer nuclear layer; OPL, outer plexiform layer; INL, inner nuclear layer; IPL, inner plexiform layer; GCL, ganglion cell layer; NFL, nerve fiber layer; NS, not significant. Scale bar = 40 μm. Inset scale bar = 10 μm. Statistical analysis was completed by using one way ANOVA. Data are expressed as mean ± SEM (*n* = 6). Significant values are indicated with asterisks: ^∗∗∗^*p* < 0.001.

TABLE S1 Qualitative comparison of the neurochemical labeling.
