# Supplementary figures and images for: Evidence of Synaptic and Neurochemical Remodeling in the Retina of Aging Degus
Source: Front Neurosci. 2020 Mar 18;14:161. doi: 10.3389/fnins.2020.00161 (PMC7095275; doi:10.3389/fnins.2020.00161)

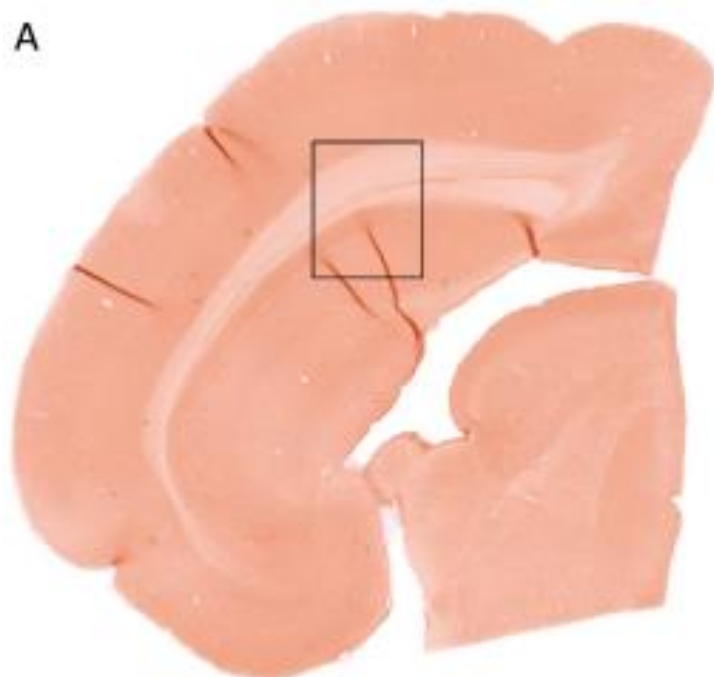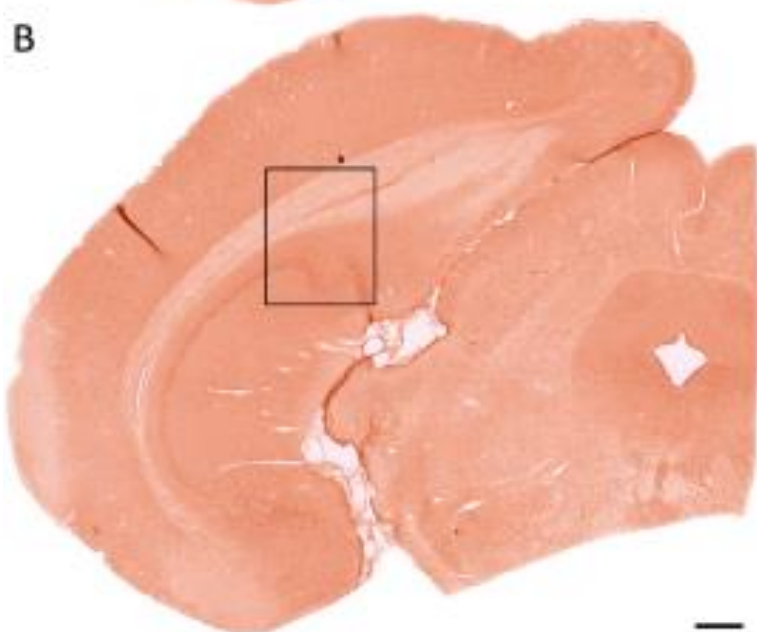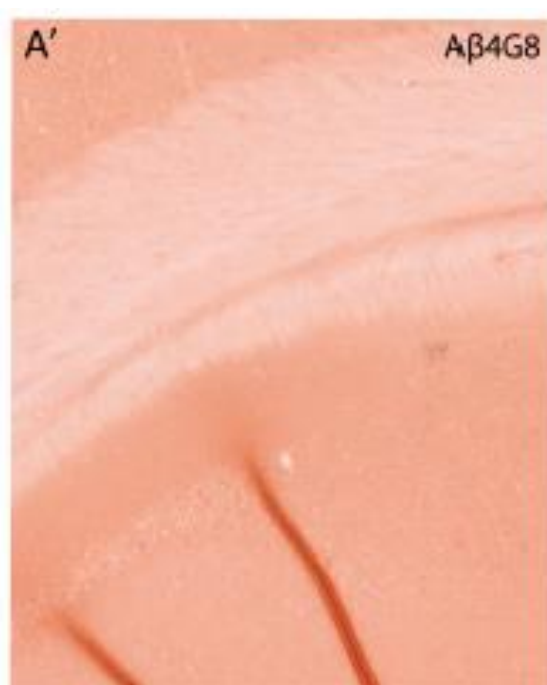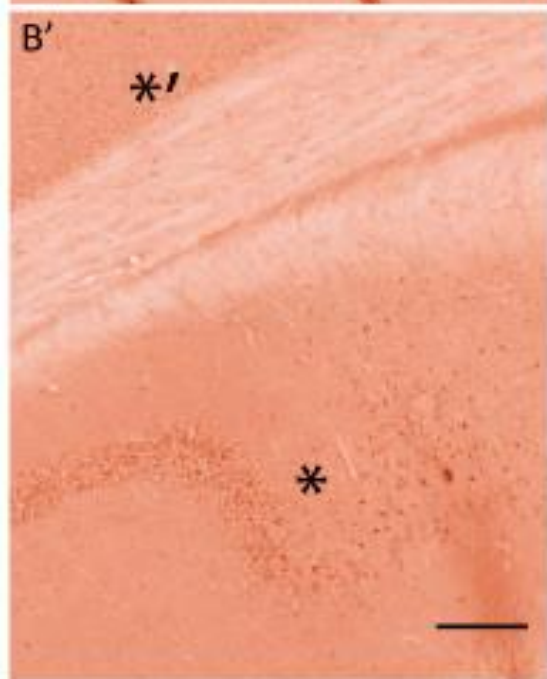

Supplement: FIGURE S1 — Aβ4G8 immuno-labeling of the degus brain. (A) No labeling in the Juvenile 5 month old degus. (A’) Magnified view of inset in (A). (B) positive labeling in the Aged Adult 96 month old degus (marked by ∗ in the hippocampus and ∗’ in the cortex). (B’) Magnified view of inset in (B’). Scale bar = 500 μm for (A,B), 200 μm for (A’,B’). [file Image_1.pdf]

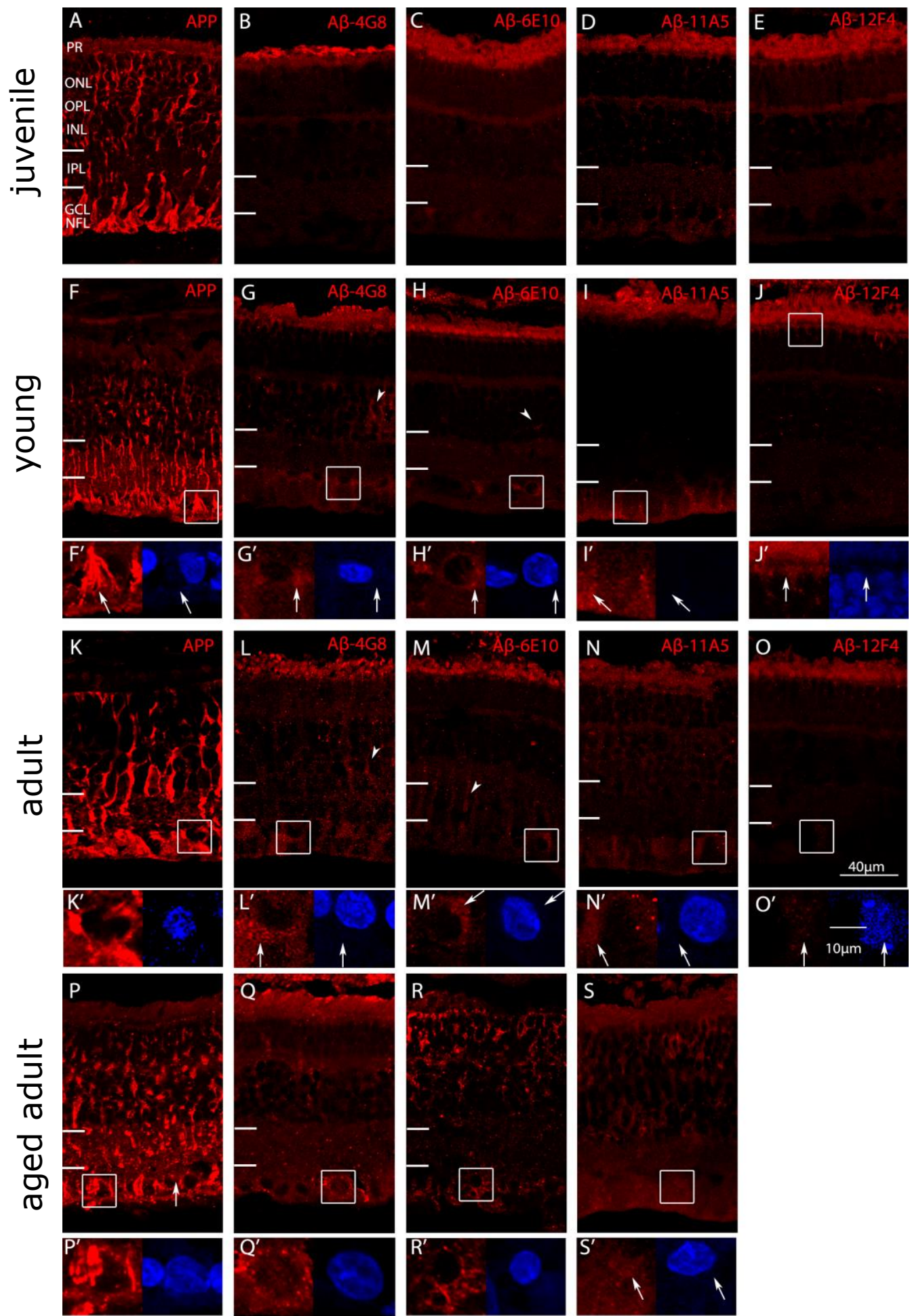

Supplement: FIGURE S2 — Expression of amyloid precursor protein (APP) (A,F,F’,K,K’P,P’); accumulation of amyloid β peptides detected with Aβ4G8 in (B,G,G’L,L’,Q, Q’), Aβ6E10 in (C,H,H’, M, M’,R,R’), Aβ11A5 in (D,I,I’, N, N’, S, S’) and Aβ12F4 in (E,J,J’,O,O’) as a function of development. (A–E) Juvenile degus, (F–J) young degus, (K–O) adult degus, (P–S) aged adult degus. APP A4 (clone 22C11) is a mouse antibody, IgG1 raised against amino acids 66–81 in the N terminus of human APP (1:100 Abacus-als, MAB348). Aβ4G8 is a mouse antibody, IgG2b raised against amino acids 17–24 of human Aβ peptide (1:300 Huntingtree SIG-39220). Aβ6E10 is a mouse antibody IgG1 against amino acids 1–16 of human Aβ peptide (1:300 Huntingtree SIG-39320). Amyloid β, clone 11A5-B10 is a mouse antibody IgG against amino acids 3–40 of human Aβ (1:100 Millipore 05-799). Amyloid β12F4 is a mouse antibody IgG1 against amino acids x-42 of human Aβpeptide (1:500 Covance SIG-39142). [file Image_2.pdf]

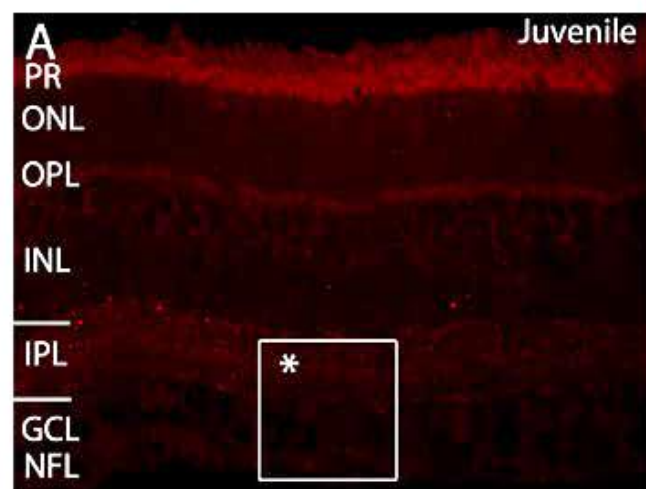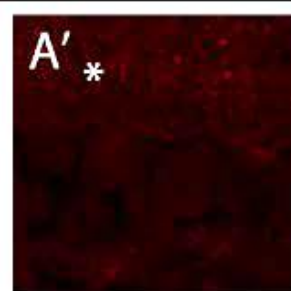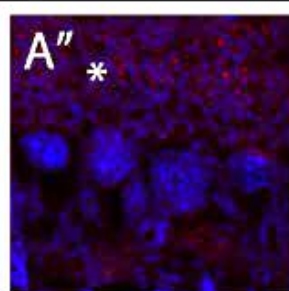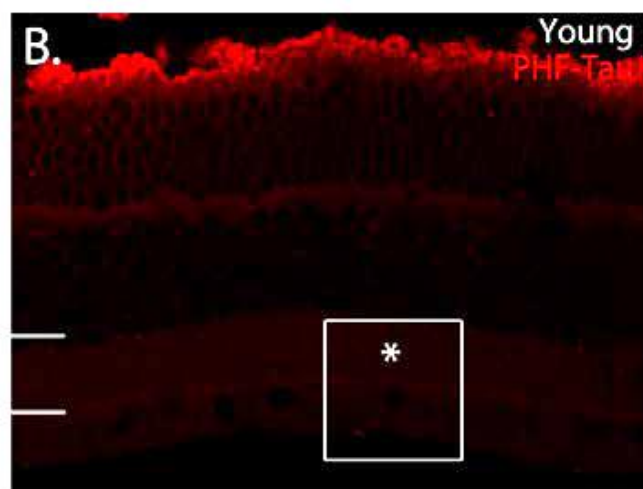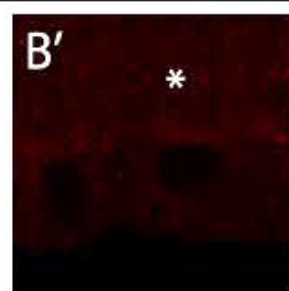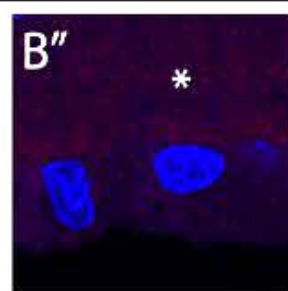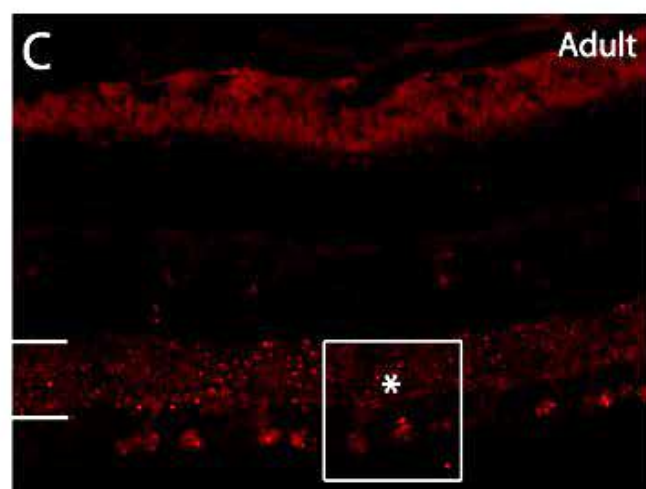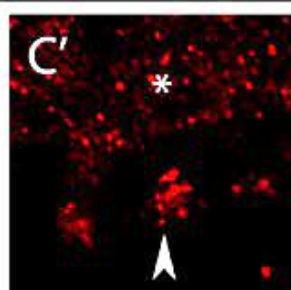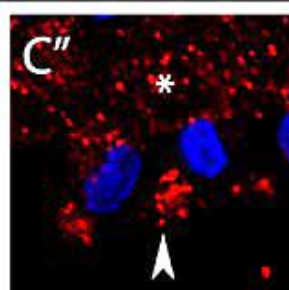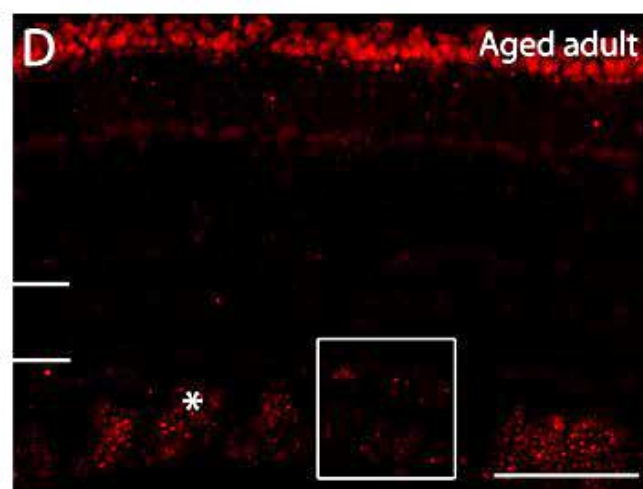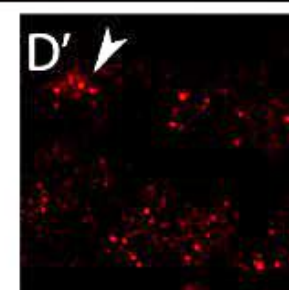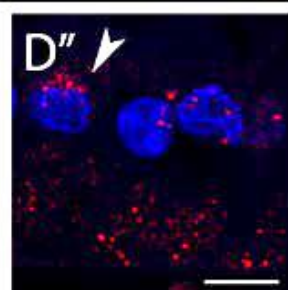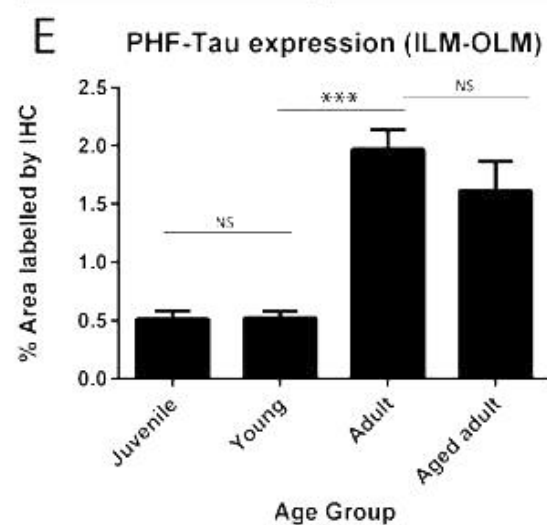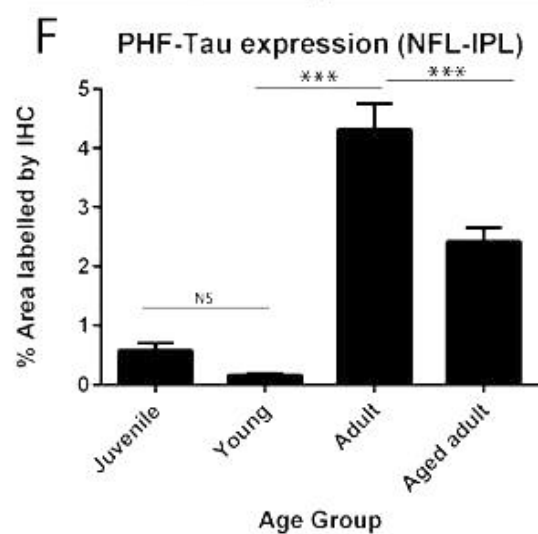

Supplement: FIGURE S3 — PHF-Tau (AT8) immuno-labeling in the degus retina. (A) Juvenile degus, (B) young degus, (C) adult degus, (D) aged adult degus. The ∗ indicates punctate labeling in the IPL and arrowheads indicate cytoplasmic deposits. (A’–D’) Magnified view for PHF-Tau corresponding to each age inset. (A”–D”) Magnified view for PHF-Tau + DAPI. (E) Quantification of the % area occupied by PHF-Tau in total retina (ILM-OLM). (F) Quantification of % area occupied by PHF-Tau in the NFL-GCL in all groups. PHF-tau (clone AT8) is a mouse antibody, IgG1 raised against phosphorylated Ser202/Thr205 in human PHF-tau (1:200 Thermo Fisher MN1020). Abbreviations: PR, photoreceptors; ONL, outer nuclear layer; OPL, outer plexiform layer; INL, inner nuclear layer; IPL, inner plexiform layer; GCL, ganglion cell layer; NFL, nerve fiber layer; NS, not significant. Scale bar = 40 μm. Inset scale bar = 10 μm. Statistical analysis was completed by using one way ANOVA. Data are expressed as mean ± SEM (n = 6). Significant values are indicated with asterisks: ∗∗∗p < 0.001. [file Image_3.pdf]
