# Supplementary material for: Evidence of Synaptic and Neurochemical Remodeling in the Retina of Aging Degus
Source: Front Neurosci. 2020 Mar 18;14:161. doi: 10.3389/fnins.2020.00161 (PMC7095275; doi:10.3389/fnins.2020.00161)
Supplement: TABLE S1 — Qualitative comparison of the neurochemical labeling. [file Table_1.pdf]

Supplementary table 1. Qualitative comparison of the neurochemical labeling.

		GC	IPL	AC	MC	BC	HC	OPL	ONL
Glutamate	Juveniles	+	+	+	-	+	+	+	+
	Young	+	++	++	+	+++	++	++	++
	Adults	+	++	+	-	+	+	+	+
	Old Adults	+++	+	+	-	++	+	+	+
Glutamine	Juveniles	+	+	+	+	+	+	+	+
	Young	-	+	+	+	++	++	+	+
	Adults	-	+	+	+	+	++	+	+
	Old Adults	++	++	++	+	++	++	++	++
GABA	Juveniles	+	+	+	-	-	-	-	-
	Young	+	+	+	-	-	+	+	-
	Adults	++	++	++	+	+	-	-	-
	Old Adults	++	+	++	+	+	+	+	+

Relative labelling of the neurochemicals comparated to the juvenile age: presence (+), not present (-) twice or more the value (++). Abbreviations: GC, ganglion cells; IPL, inner plexiform layer; AC, amacrine cell, MC, Muller cell; BC, bipolar cell; HC, horizontal cell; OPL, outer plexiform layer; ONL, outer nuclear layer
